# Effects of biochar and alkaline amendments on cadmium immobilization, selected nutrient and cadmium concentrations of lettuce (*Lactuca sativa*) in two contrasting soils

**DOI:** 10.1186/s40064-016-2019-6

**Published:** 2016-03-31

**Authors:** Desta Woldetsadik, Pay Drechsel, Bernard Keraita, Bernd Marschner, Fisseha Itanna, Heluf Gebrekidan

**Affiliations:** School of Natural Resources Management and Environmental Sciences, Haramaya University, 138, Dire Dawa, Ethiopia; International Water Management Institute, Colombo, Sri Lanka; Department of Global Health, University of Copenhagen, Copenhagen, Denmark; Department of Soil Science/Soil Ecology, Ruhr-University Bochum, Bochum, Germany; Department of Crop Science, University of Nambia, Windhoek, Namibia

**Keywords:** Biochar, Faecal matter, Cadmium, Immobilization, Phytoavailability, Lettuce

## Abstract

To assess the efficiency of seven treatments including biochars produced from dried faecal matter and manures as stabilizing agents of cadmium (Cd)-spiked soils, lettuce was grown in glasshouse on two contrasting soils. The soils used were moderately fertile silty loam and less fertile sandy loam and the applied treatments were 7 % w/w. The reduction of bioavailable Cd (ammonium nitrate extractable) and its phytoavailability for lettuce were used as assessment criteria in the evaluation of stabilization performance of each treatment. Moreover, the agronomic values of the treatments were also investigated. Ammonium nitrate extraction results indicated that faecal matter biochar, cow manure biochar and lime significantly reduced bioavailable Cd by 84–87, 65–68 and 82–91 %, respectively, as compared to the spiked controls. Unpredictably, coffee husk biochar induced significant increment of Cd in NH_4_NO_3_ extracts. The immobilization potential of faecal matter biochar and lime were superior than the other treatments. However, lime and egg shell promoted statistically lower yield and P, K and Zn concentrations response of lettuce plants compared to the biochar treatments. The lowest Cd and highest P tissue concentrations of lettuce plants were induced by faecal matter and cow manure biochar treatments in both soils. Additionally, the greatest Cd phytoavailability reduction for lettuce was induced by poultry litter and cow manure biochars in the silty loam soil. Our results indicate that faecal matter and animal manure biochars have shown great potential to promote Cd immobilization and lettuce growth response in heavily contaminated agricultural fields.

**Electronic supplementary material:**

The online version of this article (doi:10.1186/s40064-016-2019-6) contains supplementary material, which is available to authorized users.

## Background

Excessive accumulation of heavy metals in agricultural soils, lead to elevated metal uptake by crops and thus affect food quality and safety, which pose major public health concern (Wang et al. 2005; Khan et al. 2008). The potential toxicity and persistent nature of heavy metals make the process of remediating contaminated soil very complex (Wu et al. 2004). A number of ex situ remediation options are available for contaminated soils including soil washing, excavation and electrokinetics (Virkutyte et al. 2002; Yeung and Hsu 2005; Dermont et al. 2008; Peng et al. 2009). However, most of these remediation options are expensive and damages soil quality (Mulligan et al. 2001; Alkorta et al. 2004; Ghosh and Singh 2005).

In situ chemical immobilization technologies are the best demonstrated and promising alternatives to ex situ remediation methods (Diels et al. 2002; Kumpiene et al. 2008; Chen et al. 2015; Hmid et al. 2015). Chemical immobilization is based on alteration of contaminant and soil characteristics by the addition of stabilizing agents. Numerous amendments including clay minerals, organic and liming materials and phosphate minerals have been widely examined for reducing metal mobility and availability in heavy metal contaminated soils (Chen et al. 2000; Cao et al. 2003; Ok et al. 2010; Herath et al. 2015; Puga et al. 2015). The immobilization process is influenced by various mechanisms including adsorption, specific binding of metal ions, cation exchange, precipitation and complexation (Polo and Utrilla 2002; Ok et al. 2007; Uchimiya et al. 2010; Herath et al. 2015; Hmid et al. 2015).

Biochar has many heavy metal immobilization properties including microporous structure, active functional groups, high pH and cation exchange capacity (CEC) (Chen and Lin 2001; Jiang et al. 2012a, b). Biochar, originated from plant residues, have been applied to soils for immobilization of heavy metal contaminants (Chun et al. 2004; Mohan et al. 2007). In addition, Phosphorous (P)—rich biochars have also shown great potential to reduce the mobility and availability of metals in water and soils contaminated with heavy metals (Cao et al. 2009a; Uchimiya et al. 2010). Accordingly, biochars derived from animal wastes have been spotlighted as heavy metal stabilization agents in contaminated soils (Cao and Harris 2010; Cao et al. 2011; Park et al. 2011a). Alkaline amendments also used as immobilizing agents in contaminated soil may have profound effects on reducing metal solubility and mobility via increasing soil pH and concomitantly metal sorption to soil particles and formation of poorly soluble metal hydroxides and carbonates (Filius et al. 1998; Kumpiene et al. 2008; Zeng et al. 2011). Recently, lime-based waste materials have been assessed for their potential to stabilize heavy metals and highlighted as an environmentally friendly immobilization approach (Ok et al. 2010, 2011a, b; Lee et al. 2013).

Although the immobilization of heavy metals using various organic and inorganic amendments including plant and animal derived biochars and lime-based materials have been well studied (Chun et al. 2004; Ok et al. 2010; Uchimiya et al. 2010; Liu et al. 2009; Lee et al. 2013), little is known about the potential human faecal matter (FM), *Prosopis juliflora* pods (PJ) and coffee husk (CH) biochars in reducing the mobility and bioavailability of heavy metals in contaminated soils. Therefore, the objective of this study was to evaluate efficacy of biochars [FM, PJ, CH, cow manure (CM) and Poultry litter (PL)] and alkaline amendments [egg shell (ES) and lime (LI)] as stabilizing agents of Cd in spiked soils. The efficacy of immobilization was evaluated by the change in Cd concentration in NH_4_NO_3_ extract and phytoavailability of the metal for lettuce.

## Methods

### Soil and feedstock sampling and preparation

Soils of two different texture classes i.e. silty loam (PK) and sandy loam (BA), were collected for greenhouse experiments from two sites i.e. a wastewater irrigated urban vegetable farming site in Addis Ababa and a rainfed peri-urban groundnut farming site in Babile, Ethiopia. At each site, approximately 100 kg of composite soil sample was excavated from the surface to a depth of 15 cm. The soil samples were transported to the greenhouse in plastic bags. The samples were air-dried, homogenized, and sieved using a <2 mm sieve.

### Stabilization treatments

Faecal matter (FM) was collected from septage drying bed in Addis Ababa sewage treatment plant. Samples were taken from 12 different locations at 10 cm depth, then mixed into one composite sample. Poultry litter (PL) was also obtained from drying bed in a commercial deep—bedded poultry farm in Bishoftu. Cow manure (CM) was collected from a private milking facility. *Prosopis juliflora* (PJ) pods were collected from different *Prosopis juliflora* invaded lands in a peri-urban area of Dire Dawa. Coffee husk (CH) was also collected from raw coffee processing facility in Addis Ababa. Cow manure samples underwent air-drying in a glasshouse for 10 days.

For pyrolysis, the feedstock samples were placed in aluminum furnace (FATALUMINUM S.p.A, ITALY). The heating rate was 15 ^◦^C/min. Heat treatments were performed at 450 ^◦^C for FM, CM and PL, 480 ^◦^C for PJ and 375 °C for CH. The pyrolysis temperature was maintained for 60 min for FM, CM and PL, for 62 min for PL and for 55 min for CH. After pyrolysis, the charred samples were removed from the canister and allowed to cool to room temperature. The egg shell powder (ES) was also prepared with waste egg shells collected from ELFORA plc in Bishoftu. The egg shells were washed several times with hot water, then heated at 72 °C for 72 h to dry, subsequently pulverized using a mortar and pestle to homogenized powder having <1 mm particle size (Ok et al. 2011a). Lime (LI) was also obtained from National Soil Testing Center.

### Experimental set-up

Experimentation was done in a greenhouse. The treatments used in this study were FM, CM, PL, PJ and CH biochars, ES and LI. Cadmium was applied to soil as solution of cadmium (II) nitrate tetrahydrate (Cd(NO_3_)_2_.4H_2_O) at the rate of 50 mg Cd/kg. Treatments were homogenized with Cd spiked soils at the rate of 7 % w/w. Briefly, 3 kg of air-dried Cd treated soil was thoroughly mixed with each treatment in plastic pot. For each soil type, separate trial was conducted in a completely randomized design in triplicates. The trial was carried out in a temperature controlled glasshouse with regular daily watering. After 2 weeks, eight seeds of lettuce were sown in each pot and lettuce seedlings were thinned to three per pot a week after emergence (only 3 or 4 seedlings were emerged in the control and some treatments). Pots were placed on plastic saucers to prevent leachate drainage. Ten weeks after sowing, the above ground biomass was cut down to soil surface to determine shoot fresh weight. The above ground biomass was cleaned to avoid the adhered soil particles. Dry weight was subsequently determined following oven drying to a constant weight at 65 °C for 72 h. The dried lettuce plants were ground, milled to fine powder and stored for subsequent analyses. After harvesting, soil sample from each pot was collected, ground to <2 mm and stored for pH and NH_4_NO_3_ extractable Cd analyses. Phytoavailability was computed as follows (Cao et al. 2009b).

$${\text{Phytoavailability}}\,\left( \% \right) \, = \, \frac{{{\text{metal concentration}}\left( {{\text{mg}}/{\text{kg}}} \right){\text{ in plant }} \times {\text{ above ground biomass}}\left( {{\text{kg}}/{\text{pot}}} \right)}}{{{\text{metal concentration}}\left( {\text{mg/kg}} \right){\text{ in soil }} \times {\text{ soil mass }}\left( {\text{kg/pot}} \right)}}$$

## Analyses

First, the soil and biochar samples were ground to <2 mm. For total element, NH_4_NO_3_ extractable trace elements and Fourier Transform Infrared (FTIR) analyses, soils and biochar samples were milled with a planetary ball mill to achieve a homogeneous fine powder (Fritsch GmbH, Idar-Oberstein, Germany). The pH of biochar in water was determined in 1:20 (w/v) ratio after occasionally stirring over 1 h (Cheng et al. 2006). The pH of the soils in water suspensions were determined in 1:2.5 (w/v) ratio after shaking over 2 h. The EC of biochar was determined after 1 h equilibration of 1 g of biochar with 20 ml of distilled water. The EC of the soil was determined after 2 h equilibration of 1 g of soil with 2.5 ml of distilled water. Soil Particle size distributions were determined by laser diffraction using an Analysette 22 MicroTec plus (Fritsch GmbH, Idar-Oberstein, Germany) with a wet dispersion unit. For total element analysis, 0.25 g of biochar and soil were placed into 50 ml vessels, followed by addition of 10 ml concentrated HNO_3_. The mixtures were left to cold digest in a fume cupboard over night and then heated in 1.6 KW microwave oven for 30 min. After cooling to room temperature, 10 ml of double distilled water was added into the vessel and filtered via a 0.45 µm cellulose nitrate filter paper. Finally, the filtrate was subjected to the total element analysis using ICP-OES (Ciros CCD, SPECTRO Analytical Instruments GmbH, Kleve, Germany). Olsen-P (available P) were extracted by placing 1 g of soil and biochar in 20 ml of NaHCO_3_ for 30 min. The suspension was vacuum filtered via a 0.45 µm cellulose nitrate filter paper and analyzed using ICP-OES (Ciros CCD, SPECTRO Analytical Instruments GmbH, Kleve, Germany). For C and N analyses, about 3.5 mg of biochar and 40 mg of soil were weighted into sample boats and determined using C and N analyzer (Elementar Analyse GmbH, Hanau, Germany). Acetanilide was used as calibration standard. Total surface acidity was determined by adding 0.15 gm of biochar into 15 ml of 0.1 N NaOH and shaken for 30 h. The suspension was vacuum filtered and 5 ml of 0.1 N NaOH aliquot was transferred to 10 ml of 0.1 N HCl to completely neutralize the unreacted base. The solution was back-titrated with 0.1 N NaOH using a Metrohm 725 Dosimat (Metrohm AG, Herisau, Switzerland) fitted with a 691 pH meter (Metrohm AG, Herisau, Switzerland). Similarly, the surface basicity was measured by shaking 0.15 g of biochar with 15 ml of 0.1 N HCl for 30 h. The slurry was vacuum filtrated (0.45 µm) and an aliquot of 5 ml of 0.1 N HCl was mixed with 10 ml of 0.1 N NaOH to neutralize the unreacted acid. The solution was back-titrated with 0.1 N HCl. The total surface acidity and basicity were determined by calculating the base and acid uptake of biochars (Goertzen et al. 2010). For dissolved organic carbon (DOC) determination, extract was prepared by shaking biochar with 0.01 M CaCl_2_ at 1:25 ration (w/v) for 1 h. The suspension was vacuum filtered and measured by a Dimatoc 2000 (DIMATEC Analysentechnik GmbH, Essen, Germany). The exchangeable cations and CEC of biochar were determined using BaCl_2_ method. Briefly, 2.5 gm of biochar was weighted into 50 ml centrifuge tube, followed by addition of 30 ml of 0.1 M BaCl_2_. The tube was shaken for 1 h and then centrifuged at 5500 rpm for 10 min. After centrifugation, the supernatant was decanted into a 100 ml volumetric flask. This procedure was repeated three times. The collected supernatants were made up to 100 ml with 0.1 M BaCl_2_ solution. The Na, Mg, Ca, K and Al concentrations of the solution were determined using ICP-OES (Ciros CCD, SPECTRO Analytical Instruments GmbH, Kleve, Germany). The same procedure was followed to determine the water soluble Na, Mg, Ca, K and Al concentrations of biochar. Finally, the concentration of exchangeable cations and CEC of biochar was computed by subtracting the concentration of water soluble cations (Na, Mg, Ca and K) to the concentration of cations extracted by 0.1 M BaCl_2_. For FTIR analyses of biochars, pellets were prepared by mixing biochars with KBr powder and then analyzed using a Tensor 27 FTIR Spectrometer (Bruker optik GmbH, Ettlingen, Germany). Spectra were collected in the range of 400–4000 cm^−1^ at 4 cm^−1^ and 120 scans per sample. Surface areas of the biochars were determined using adsorption data of the adsorption isotherms of N_2_ at −196  °C and calculated by the Brunauer–Emmet–Teller (BET) equation (Brunauer et al. 1938). For biochar and post harvest soil samples, NH_4_NO_3_ (1 M) extractable fraction of Cd was determined following the extraction procedure proposed by the German national standard (DIN 19730 2009). A milled plant sample was analyzed for total Cd, P, K, Ca, Mg and Zn concentrations as previously described.

### Statistical analysis

Data are presented as mean (standard deviation) and were computed using Microsoft 2007 excel software. Treatment effects were determined by analysis of variance according the general linear model procedure of SAS. Different among means of treatment effects were separated by least significant difference (LSD) at *P* < 0.05 using SAS 9.2 software.

## Results and discussion

### Characterization of soils and stabilization treatments

Table 1 shows selected properties of PK and BA soils. PK soil was silty loam having pH 6.71(H_2_O) and relatively high in exchangeable cations compared to BA soil. The pH(H_2_O) of BA soil was 6.86 with a sandy loam texture. The total Cd concentration of PK soil (2.58 mg/kg) was higher than BA soil (0.30 mg/kg). Soil carbon status of PK soil was rated as moderate, whereas soil carbon concentration of BA soil was rated as very low according to Tekalign (1991). Similarly, BA soil had a low total N content as compared to the critical concentration reported in Peverill et al. (1999).

**Table 1:** Selected properties of the soils

Soil	pH (H_2_O)	EC (dS/m)	Exchangeable cations [cmol_(+)_/kg]					CEC [cmol(+)/kg]	Total Cd (mg kg^−1^)	Total C (g kg^−1^)	Total N (g kg^−1^)	Particle size		
			Ca	Mg	K	Na	Al					% Sand	% Silt	% Clay
PK^a^	6.71	0.024	24	6.7	0.9	0.4	<0.02	32.2	2.58	19	1.8	19.1	73.6	7.2
BA^a^	6.86	0.006	4.2	1.1	0.3	0	<0.02	5.83	0.3	3.2	0.4	54.1	38.2	7.5

^a^PK soil: Silty loam soil; BA soil: Sandy loam soil

In contrast to the more alkaline pH(H_2_O) of CM, PJ and PL biochars, biochars from CH and FM had slightly alkaline pH values (Additional file 1: Table S1). ES also had high pH value of 9.28 and contained considerable amount of calcite (CaCO_3_) (Lee et al. 2013). Similarly, biochars produced from PJ, PL and CM had high EC values, whereas, CH and FC biochars exhibited low EC values. These were expected considering the high salt/ash content in CM and PL biochars (Cantrell et al. 2012). The biochar treatments had varied total C concentration, with FM < CM < PL < PJ < CH. Unlike CH biochar which exhibited the highest concentration of total C and the lowest concentration of total N typical feature of plant-based biochars (Gaskin et al. 2008; Singh et al. 2010), the other biochar treatments including PJ had very high concentrations of total N (Additional file 1: Table S1). Moreover, CH biochar had the highest surface area (206 m^2^/g). The total surface acidity of the examined biochar treatments ranged from 0.42 to 3.24 mmol/g (Additional file 1: Table S1). The acidic surface functionality might caused by the presence of carboxyl, phenolic and lactonic groups. Whereas, ketones, carbonates and other alkaline species might be responsible for basic surface functionality (Mukerjee et al. 2011). With the exception of CH biochar, total acidic surface functionalities of the biochar treatments were less than their corresponding basic functionalities. These observations were consistent with the study of Singh et al. (2010), who recorded high total surface basicity than surface acidity in PL and CM biochars produced at 550 °C with steam activation and Uras et al. (2012) who reported high surface acidity than surface basicity in plant based biochars.

Although faecal matter and manure derived biochar treatments had high concentrations of total P and major cations, the total P, Fe, Al and Mg concentrations in FM biochar were higher than the concentrations in other biochar treatments (Additional file 1: Table S2). Yet again, the FM biochar had the highest total trace elements. However, CM and PJ biochars contained the highest concentrations of Ca (34 g kg^−1^) and K (39.2 g kg^−1^), respectively. The high levels of P, K, Mg and Ca in the biochars were consistent with the results of Song and Guo (2012), who reported very high concentrations of these elements in PL biochars produced under various pyrolysis temperatures. The highest exchangeable K (59.6 cmol_(+)_ kg^−1^) was observed in PJ biochar, while the lowest (1.60 and 1.61 cmol_(+)_ kg^−1^) were recorded in CH and FM biochars, respectively. However, CM and PL biochars exhibited the highest exchangeable Mg and Ca concentrations, respectively. Generally, the CEC of the biochar treatments were in the order of PJ > CM > PL > FM > CH (Additional file 1: Table S3). In comparison, the CEC of PL biochar was 12.2 % higher than similar biochar with an average value of 37 cmol_(+)_ kg^−1^ despite the fact that the methods of CEC measurement differed (Song and Guo 2012). There were also differences in Olsen-P (available P) concentrations of biochar treatments, with CMB > FMB > PLB > PJ > CH (Additional file 1: Table S3). As expected plant-based biochar treatments exhibited the lowest Olsen-P values of 28.1 mg kg^−1^ (CH) and 383 mg kg^−1^ (PJ). On the contrary to the total P, the highest available P (1437 mg kg^−1^) was exhibited by CM biochar. Likewise, the study of Cao and Harris (2010) showed very high water soluble P value of CM biochar produced under very low pyrolysis temperature. Available P value of FM biochar decreased to higher degree to its corresponding total P value, this was largely ascribed to the formation of stable P containing compounds.

Biosolids are known to contain high total concentrations of trace and toxic elements, which exist in more pronounced concentrations in charred product (Bridle and Pritchard 2004; Lu et al. 2013). However, the use of biochar from biosolid is highly limited by the bioavailability nature of the trace and toxic elements than the total load. Ammonium nitrate extractable fraction was used to estimate the bioavailability of heavy metals in the examined biochar treatments. The mobile fractions of the metals in the biochar treatments accounted very small portion of their corresponding total contents. For example, for FM biochar treatment the bioavailable fractions were 0.83, 0.14, 0.03, 0.03, 0.04, 0.005 and 0.04 % of the total loads of Cd, Co, Cr, Cu, Ni, Pb and Zn, respectively (Additional file 1: Tables S2, S3). Overall, the bioavailable fractions in the biochars were in the range of 0.47–2.5, 0.14–0.85, 0.02–0.09, 0.015–0.11, 0.04–0.71, 0.005–0.76 and 0.04–7.76 % of the total loads of Cd, Co, Cr, Cu, Ni, Pb and Zn, respectively (Additional file 1: Tables S2, S3). FTIR spectra of FM and PL biochars were very similar (Additional file 1: Figure S1). The characteristics broad bands at 3419, 3442,3419, 3431 and 3466 cm^−1^ were attributed to the stretching vibrations of hydrogen-bonded hydroxyl groups of FM, PL, CM, PJ and CH biochars, respectively (Keiluweit et al. 2010). For all biochars, but CHB, aromatic C=C ring stretching were observed between 1462 and 1433 cm^−1^.The presence of C=O stretching vibrations (1700–1600) indicated the presence of carboxylic groups and ketones. Considering the high P contents of faecal and manure derived biochars, particularly FMB and CMB, the intense broad bands at 1038 cm^−1^ likely resulted from P-containing functional groups, most importantly, P-O bond of phosphate functional group (Jiang et al. 2004).

### Effect of treatments on soil pH and growth of lettuce

As presented in Table 2, all stabilization treatments but CHB significantly increased soil pH over the spiked control in PK soil. In BA soil, addition of FMB had non-significant effect on soil pH, whereas all other treatments significantly increased the pH of the soil compared to the spiked control. Similar to this study, the findings of several studies indicated that the application of biochar and alkaline amendments enhanced soil pH (Chan et al. 2007, 2008; Lee et al. 2008; Ok et al. 2011a, b). Among the stabilization treatments, LI and ES promoted the greatest pH increase in both soils, mainly due to the alkaline impact of LI and ES (lime-based material) containing CaCO_3_, which dissociate to Ca^2+^ and CO_3_^2−^, consequently the reaction of CO_3_^2−^ with water liberate OH^−1^ ions, thereby resulting in soil pH increase (Ok et al. 2011a; Lee et al. 2013).

**Table 2:** The influence of stabilization treatments on pH and fresh weight (FW) shoot yield of lettuce grown on PK and BA soils

Stabilization treatments	PK^a^ soil		BA^a^ soil	
	Soil pH	Shoot yield (FW)	Soil pH	Shoot yield (FW)
FMB	7.04 (0.09)e^b^	144 (13.0)a	8.39 (0.09)bc	91.5 (3.69)a
CMB	7.02 (0.06)e	84.2(2.65)c	8.83 (0.25)a	37.6 (0.40)b
PLB	6.89 (0.04)f	25.1 (2.78)g	8.48 (0.20)b	20.5 (1.87)e
PJB	7.18 (0.10)d	31.8 (2.40)g	8.83 (0.39)a	24.0 (1.72)d
CHB	6.58 (0.03)g	55.9 (2.19)e	8.46 (0.14)b	31.1 (0.81)c
ES	7.74 (0.08)b	61.0 (1.90)e	8.88 (0.02)a	13.2 (1.34)f
LI	7.95 (0.05)a	43.2 (1.89)f	9.09 (0.23)a	10.0 (0.96)g
CON^+^	6.51 (0.04)g	73.1 (3.57)d	8.11 (0.04)c	12.7 (0.36)fg
CON^−^	7.41 (0.02)c	106 (6.77)b	8.17 (0.06)bc	30.8 (2.70)c

*FMB* Faecal matter (Faecal cake) biochar, *CMB* cow manure biochar, *PLB* poultry litter biochar, *PJB* *Prosopis juliflora* pods biochar, *CHB* Coffee husk biochar, *ES* Eggshell waste, *LI* Lime, *CON* ^*+*^ spiked control, *CON* ^*−*^ non-spiked control

^a^PK soil: Silty loam soil; BA soil: Sandy loam soil

^b^Standard deviation in parentheses (n = 3), values for each soil with different letter within each column are significantly different (*P* < 0.05)

With the exception of FMB and CMB, all other stabilizing treatments induced significant shoot yield reduction of lettuce plants grown in PK soil compared to the spiked control (Table 2). Faecal matter biochar promoted significant shoot yield response of lettuce plants, 97 %, compared to the spiked control. Likewise, a more profound effect of FMB application in increasing shoot yield of lettuce plants as high as 620 % was also observed in BA soil. The positive impact of FMB on the growth performance of lettuce, compared to the controls, may be attributed to a combination of P nutrition and toxicity reduction effects (Chen et al. 2006). This was evident from the high P concentration of lettuce plants under this amendment. In agreement with this finding, applying biochar from sewage sludge significantly improved garlic yields even at lower biochar-to-soil ratios (Song et al. 2014). Moreover, significant increase in shoot yield of lettuce plants grown in BA soil was also observed across all other biochar treatments, increasing by 196, 61, 89, and 145 % under CMB, PLB, PJB and CHB, respectively. Similarly, Park et al. (2011a) and Karami et al. (2011) reported improved dry matter yield of Indian mustard and ryegrass plants grown in heavy metal contaminated/spiked soils treated with CM and green waste biochar, respectively, compared to no amendment control, suggesting the potential of biochar to enhance fertility of soil and reduce phytotoxicity of the metals. Conversely, the addition of wood biochar to Cd spiked soil (sandy) didn’t promote significant dry matter yield effect of maize (Namgay et al. 2010). Meanwhile, the results of this study showed that the shoot yield of lettuce plants was significantly decreased as a result of Cd spiking as compared to the non-spiked control, indicating phytotoxicity of Cd to lettuce plants.

### Ammonium nitrate extractability of Cd

The results of this study revealed significant effect of biochar and alkaline treatments, not including CHB, on reducing NH_4_NO_3_ extractable Cd in both soils (Table 4). Compared to the spiked controls, NH_4_NO_3_ extractability of soil Cd decreased by 50–88 % under ES treatments. Similarly, the concentrations of Cd in NH_4_NO_3_ extracts were reduced by 70–85 % under PLB treatment compared to the spiked controls. Comparatively, FMB and LI promoted statistically the greatest decrease in concentrations of NH_4_NO_3_ extractable Cd in both soils (1.15–1.97 mg/kg DW). However, PJB and CMB exerted significant, but relatively smaller, reduction in NH_4_NO_3_ extractable Cd (32–67 and 65–68 %), respectively. In field study using LI as a treatment, Gray et al. (2006) reported significant reduction of Cd concentration in NH_4_NO_3_ extract. In previous study, Uchimiya et al. (2010) recorded significant immobilization Cd in contaminated soil amended with manure derived biochars. Very recently, Hmid et al. (2015) has reported considerable reductions of Ca(NO_3_)_2_ extractable Cd and other metals with increasing rates of olive mill waste biochar. Immobilization of Cd and other heavy metals by biochar and alkaline treatments induced by enormous mechanisms including ion exchange, electrostatic interaction, surface complexation, precipitation of amorphous to poorly crystalline metal phosphate minerals, substitution for Ca by Cd during co-precipitation (Cao et al. 2009a; Uchimiya et al. 2010; Beesley et al. 2011; Uchimiya et al. 2011b). However, it is not easy to quantify specific immobilization mechanism and it appears that the combined effect of two or more mechanisms leads to metal stabilization (Cao et al. 2003). Heavy metal immobilization by alkaline amendments is mainly attributed to soil pH rise, which increase negatively charged sites on soil particles and consequently promote cationic metal adsorption (Bradl 2004; OK et al. 2007). Moreover, Cd precipitate as Cd(OH)_2_ is highly probable at pH value above 8 (Lee et al. 2008). Several studies, Hong et al. (2007), Ok et al. (2011b) and Ahmad et al. (2012), also used lime-rich materials including those employed in our study to reduce the mobility and bioavailability of heavy metals in contaminated soils. Among the biochar treatments, application of FMB didn’t significantly affect the pH of the spiked soil consequently reduction of Cd bioavailability may not induced by the pH change in BA soil. The application of FMB most effectively reduced NH_4_NO_3_ extractable Cd by 84–87 %, while PJB showed the least decrease compared to the spiked controls. Owing to the low SSA of FMB than the other manure derived biochar treatments (Additional file 1: Table S1), this observation wasn’t related to the surface adsorption. Likewise, rice straw biochar promoted the greatest Cd stabilization effect in soils had the lowest SSA compared to husk and bran biochars (Zheng et al. 2012). Generally, the contribution of surface adsorption to Cd stabilization was limited since PJB, PLB and FMB had very small SSA ranged from 0.79 to 3.36 m^2^/g (Additional file 1: Table S1). However, sorption of Cd to surface of CMB may not be ignored. Unlike the plant based biochars (PJ and CH), which had high % C (62–73 %), manure derived biochars (FMB, CMB and PLB) exhibited low C contents (19.5–43.4 %) with the remaining being ash. The result suggest that the ash portions of these biochars may be responsible for immobilization of Cd. Moreover, one important mechanism for the reduction of NH_4_NO_3_ extractable Cd is the formation of poorly soluble Cd phosphate precipitate via specific metal ligand complexation involving phosphate functional groups on the surface of, or released by, P-rich amendments (Chen et al. 2007; Park et al. 2011a, b). This was well supported by the presence of high Olsen—P value (Additional file 1: Table S3) and phosphate functional group in FTIR spectra of FM, CM and PL biochars (Additional file 1: Figure S1). Previous studies have also demonstrated that P-bearing materials promoted heavy metal immobilization via the formation of stable phosphate minerals in contaminated soils (Cao et al. 2003, 2009a; Uchimiya et al. 2010). Furthermore, for soils treated with FM biochar, the reduction of NH_4_NO_3_ extractable Cd may also be associated with surface complexion of the metal with active carboxyl, lactones and carbonyl functional groups, owing to the relatively high total surface acidity of this biochar as compared to the other biochar treatments. Uchimiya et al. (2011a) noted a role of cation exchange capacity in reducing chemically mobile metals under biochar amendment via the release of K, Ca, Na and Mg. This may probably occurred in soils amended with PJ, CM and PL biochars having high CEC values (Additional file 1: Table S3). One unusual observation is that CHB with high SSA did show significant increment of Cd concentrations in NH_4_NO_3_ extract by 102–115 % compared to the spiked controls. This signifies other CHB characteristics that may greatly influence the mobility of Cd in spiked soils. High bioavailable Cd concentration in spiked soils amended with CHB may be associated with the relatively high NH_4_NO_3_ extractable Zn and DOC from the biochar (Additional file 1: Tables S1, S3), with both DOC and bioavailable Zn influences the mobility and bioavailability of Cd. In agreement with our study, Smilde et al. (1992) reported significant raise of CaCl_2_ extractable Cd as a consequence of Zn application in a loam soil. Furthermore, Beesley et al. (2010) reported mobilization of Cd and Zn with increases in DOC. In earlier study, Antoniadis and Alloway (2002) also reported that DOC application raised CaCl_2_ extractability of Cd in sewage sludge amended soils. This effect may be explained in terms of displacement of Cd from the exchange complex.

### Effect of treatments on Cd and nutrient concentrations on lettuce

Compared to the spiked controls, all stabilization treatments did show significant reduction of Cd concentrations of lettuce plants (Table 3). In both soils, tissue Cd concentrations of lettuce plants grown in Cd spiked soils amended with FMB was statistically the lowest compared to the other treatments, but CMB application induced statistically comparable Cd concentration in PK soil. Moreover, PLB application also resulted in noticeable (71–83 %) reduction of Cd concentrations compared to the controls. Among the treatments, ES promoted the lowest decrease (30–64 %) in Cd concentrations. Generally, the effect of the treatments in decreasing Cd concentrations followed the order: CMB > FMB > PLB > CHB > PJB > LI > ES in PK soil and FMB > CMB > PLB > PJB > LI > CHB > ES in BA soil. Overall, these findings may imply that application of stabilizing treatments, except CHB, in spiked soils have resulted in reduction of NH_4_NO_3_ extractable Cd which was then reflected back in the decreased concentration of the metal in shoot of lettuce plants. These results were supported by the findings of other investigators (Karami et al. 2011; Park et al. 2011a; Houben et al. 2013), who reported corresponding heavy metal plant concentrations reduction as a consequence of a decrease in bioavailable metal fractions in contaminated soils treated with various amendments. Yet, reduced Cd concentration can also be attributed to dilution effect due to increasing lettuce biomass under FMB treatment. Furthermore, the reduction in Cd concentration may also be associated with the sequestration of the metal in the roots of lettuce plants grown in Cd spiked soils amended with the stated treatments, most importantly under CHB treatment, with only small parts being translocated to above ground biomass (Moreno-Caselles et al. 2000). Yang et al. (1996) found that Cd translocation to shoot of ryegrass was negligible, very high concentration retained in the root. Phosphorous concentrations were significantly elevated in lettuce plants harvested from Cd spiked soils amended with FMB and CMB as compared to the other stabilization treatments. This was in good agreement with the high available P content of these treatments (Additional file 1: Table S3). On the contrary, all biochar treatments including FMB and CMB promoted significant reduction of Ca concentrations compared to the spiked controls. Again, with the exception of FMB, all biochar treatments also induced significant decrease of Mg concentrations of lettuce plants grown in BA soil. Nevertheless, addition of alkaline amendments (ES and LI) significantly increased Ca concentrations over the other stabilizing treatments in both soils. This corresponds with the high accumulation of Ca in lime-rich materials (Ahmad et al. 2012; Ok et al. 2011b). Among all treatments, PJB promoted the greatest K concentrations increase in both soils (Table 3). Similar to this observation, Chan et al. (2007) and Gaskin et al. (2010) reported very high K concentrations of crops grown in soils amended with plant—based biochars.

**Table 3:** Shoot dry weight (DW), Cd and selected nutrient tissue concentrations (DW) of lettuce grown on spiked soils under different stabilization treatments

	FMB	CMB	PLB	PJB	CHB	ES	LI	CON^+^	*l.s.d*
PK^a^ soil									
Biomass (g/pot)	8.69 (0.25)a^b^	5.63 (0.40)b	1.83 (0.14)f	2.05 (0.08)f	3.48 (0.19)d	3.68 (0.05)d	2.79 (0.12)e	4.42 (0.24)c	0.369
Cd (mg/kg)	5.50 (0.41)f	4.65 (0.15)f	9.40 (0.72)e	15.6 (0.96)c	14.2 (1.32)d	22.7 (0.95)b	16.4 (0.40)c	32.2 (0.77)a	1.37
P (g/kg)	8.15 (0.39)a	7.38 (0.30)b	6.80 (0.29)c	5.48 (0.21)d	4.21 (0.05)e	4.10 (0.17)e	4.37 (0.20)e	5.35 (0.18)d	0.422
Ca (g/kg)	13.4 (1.18)f	14.8 (0.38)ef	20.4 (0.77)c	16.2 (1.44)d	15.9 (0.21)de	24.0 (0.63)a	22.4 (0.70)b	19.7 (0.74)c	1.457
K (g/kg)	59.6 (1.21)cd	63.1 (1.50)b	61.3 (0.88)bc	68.4 (1.64)a	59.1 (0.98)cd	58.7 (1.97)d	58.0 (2.04)d	51.6 (1.11)e	2.555
Mg (g/kg)	6.83 (0.30)ab	6.31 (0.35)b	7.29 (0.47)a	5.32 (0.14)c	4.58 (0.15)d	6.64 (0.38)b	7.24 (0.31)a	4.87 (0.18)cd	0.532
Zn (mg/kg)	215 (11.7)c	298 (15.9)b	505 (5.21)a	192 (11.1)d	174 (10.2)e	61.4 (4.14)f	51.6 (5.15)f	58.4 (0.76)f	16.1
BA^a^ soil									
Biomass (g/pot)	5.67 (0.26)a	2.41 (0.04)b	1.36 (0.05)e	1.58 (0.09)d	1.81 (0.02)c	0.98 (0.04)f	0.87 (0.03)f	0.95 (0.03)f	0.178
Cd (mg/kg)	14.4 (0.66)e	20.1 (1.88)d	21.0 (0.09)d	22.1 (1.73)cd	43.4 (2.13)b	44.6 (3.52)b	24.1 (0.35)c	125 (0.33)a	3.011
P (g/kg)	5.90 (0.17)b	6.22 (0.26)a	4.56 (0.07)c	3.48 (0.08)d	2.42 (0.15)e	1.98 (0.08)f	1.29 (0.05)g	1.80 (0.03)f	0.227
Ca (g/kg)	7.10 (0.28)f	3.56 (0.35)g	8.59 (0.94)e	7.73 (0.37)ef	12.1 (0.15)d	38.8 (1.14)a	27.7 (0.48)d	16.7 (0.71)c	1.109
K (g/kg)	38.1 (0.74)c	59.6 (1.02)b	40.0 (0.76)c	62.8 (2.20)a	35.1 (1.58)d	28.8 (0.64)e	28.8 (1.21)e	30.6 (0.81)e	2.125
Mg (g/kg)	7.75 (0.37)b	5.32 (0.36)d	6.00 (0.26)c	3.47 (0.28)f	4.23 (0.28)e	7.25 (0.10)b	13.5 (0.61)a	7.45 (0.17)b	0.579
Zn (mg/kg)	50.7 (2.26)c	54.8 (1.73)c	60.4 (2.61)c	95.7 (6.33)b	175 (0.24)a	30.5 (2.58)d	28.0 (1.43)d	53.7 (2.41)c	19.99

*FMB* Faecal matter (Faecal cake) biochar, *CMB* cow manure biochar, *PLB* poultry litter biochar, *PJB* *Prosopis juliflora* pods biochar, *CHB* Coffee husk biochar, *ES* Eggshell waste, *LI* Lime, *CON* ^*+*^ spiked control

^a^PK soil: Silty loam soil; BA soil: Sandy loam soil

^b^Standard deviation in parentheses (n = 3), values for each soil with different letter within each row are significantly different (*P* < 0.05)

### Phytoavailability of Cd for lettuce

All stabilization treatments significantly reduced phytoavailability of Cd in both soils (Table 4). In PK soil, the greatest reduction of Cd phytoavailability for lettuce was exhibited following PLB (88 %) and CMB (82 %) treatments. In the same soil, the lowest reduction of phytoavailability of Cd to the test crop was recorded under ES (41 %) amendment. Conversely, greatest reduction of Cd phytoavailability for lettuce was obtained under LI (82 %) amendment in BA soil. In comparison, biochar treatments had pronounced effect in reduction of phytoavailability of Cd for lettuce in PK than BA soil. Although FMB promoted statistically the lowest lettuce Cd concentration, the phytoavailability of the metal for lettuce under this treatment was statistically lower or similar with the other stabilization treatments. This may be explained by the fact that the reduction of lettuce Cd concentration could be offset by the biomass increase, resulting in small change in phytoavailability. Other study has also reported similar observation (Cao et al. 2009b).

**Table 4:** Effects of different stabilization treatments on NH_4_NO_3_ extractability (mg/kg) of Cd and phytoavailability for lettuce on two spiked soils

Stabilization treatments	Cd			
	PK^a^ soil		BA^a^ soil	
	NH_4_NO_3_ extractability	Phytoavailability	NH_4_NO_3_ extractability	Phytoavailability
FMB	0.339 (0.023)d^b^	0.00030 (0.000022)c^b^	0.187 (0.009)e	0.00054 (0.000049)b
CMB	0.696 (0.027)c	0.000166 (0.000009)d	0.494 (0.038)d	0.00032 (0.000026)c
PLB	0.655 (0.016)c	0.000011 (0.000011)e	0.206 (0.011)e	0.00019 (0.000007)d
PJB	0.713 (0.012)c	0.00020 (0.000004)d	0.940 (0.067)c	0.00023 (0.000009)d
CHB	4.67 (0.152)a	0.00031 (0.000044)c	2.81 (0.314)a	0.00052 (0.000030)b
ES	0.261 (0.016)de	0.00053 (0.000029)b	0.689 (0.031)d	0.00029 (0.000011)c
LI	0.198 (0.004)e	0.00029 (0.000011)c	0.245 (0.012)e	0.00014 (0.000006)e
CON^+^	2.17 (0.029)b	0.00090 (0.000032)a	1.39 (0.073)b	0.00079 (0.000027)a

*FMB* faecal matter (faecal cake) biochar, *CMB* cow manure biochar, *PLB* poultry litter biochar, *PJB* *Prosopis juliflora* pods biochar, *CHB* Coffee husk biochar, *ES* eggshell waste, *LI* lime, *CON* ^*+*^ spiked control

^a^PK soil: Silty loam soil; BA soil: Sandy loam soil

^b^Standard deviation in parentheses (n = 3), values for each soil with different letter within each column are significantly different (*P* < 0.05)

## Conclusion

All tested stabilization treatments, except CHB, have shown great potential to stabilize Cd in spiked soils, significantly reducing Cd concentration in NH_4_NO_3_ extract and phytoavailability for lettuce. However, relatively low Cd stabilization of ES, combined with low yield and nutrient concentrations response of alkaline amendments (LI and ES), make firm conclusion as to the use of faecal matter and manure derived biochars for remediation of heavy metal contaminated agricultural fields very definitive.

Generally, application of faecal matter and manure derived biochars to contaminated agricultural lands may bring multi benefits: reuse of solid waste, pathogen elimination, and stabilize heavy metals and make the soil clean and healthy which will ensure the normal growth of crops. Therefore, biochar can be potentially an attractive alternative to solve heavy metal pollution problem in urban and peri-urban farming faced by the rapid urbanization and industrialization. Nevertheless, CHB application significantly increased NH_4_NO_3_ extractable Cd, bioavailable Cd fraction in the spiked soils. Thus, unintended effect of some biochars may be potential drawbacks of its indiscriminate utilization. Moreover, immobilization technology does not alter the total heavy metal concentration in soil. Therefore, it is crucial to investigate the long-term effects of biochars on soil Cd and other heavy metal immobilization.

## Additional file

10.1186/s40064-016-2019-6 Chemical composition, surface and chemical properties of faecal matter, cow manure, poultry litter, *prosopis juliflora* pods, and coffee husk biochars.
