# Supplementary material for: Effects of biochar and alkaline amendments on cadmium immobilization, selected nutrient and cadmium concentrations of lettuce (Lactuca sativa) in two contrasting soils
Source: Springerplus. 2016 Mar 31;5:397. doi: 10.1186/s40064-016-2019-6 (PMC4816957; doi:10.1186/s40064-016-2019-6)

| Table S1. pH, Electrical conductivity (EC), total carbon (TC), total nitrogen (TN), total surface acidity (TSA), total surface basicity (TBA), BET surface area (SSA), dissolved organic carbon (DOC) of FM, CM, PL, PJ and CH biochars (average of n = 4 per char). | | | | | |
| --- | --- | --- | --- | --- | --- |
|  | FMB | CMB | PLB | PJB | CHB |
| pH(H_2_O) | 8.23 | 10.1 | 9.32 | 9.81 | 7.38 |
| pH(0.01 CaCl_2_) | 7.6 | 9.29 | 8.84 | 9.07 | 6.81 |
| EC(dS/m) | 0.34 | 1.45 | 1.46 | 2.48 | 0.13 |
| TC(g Kg^-1^) | 195 | 296 | 434 | 659 | 723 |
| TN(g Kg^-1^) | 20.2 | 13.6 | 54.6 | 24.7 | 7.81 |
| TSA(mmol/g) | 2.75 | 0.83 | 1.91 | 0.42 | 3.24 |
| TSB(mmol/g) | 4.98 | 5.79 | 4.74 | 5.37 | 0.88 |
| SSA (m^2^/g) | 3.36 | 58.5 | 1.32 | 0.79 | 206 |
| DOC (mg/l) | 5.13 | 16.4 | 121 | 26.4 | 45.2 |
| FMB: Faecal matter (Faecal cake) biochar; CMB: Cow manure biochar; PLB: Poultry litter biochar; PJB: *Prosopis juliflora* pods biochar; CHB: Coffee husk biochar | | | | | |

| Table S2. Mean value for total major and trace element (selected) concentrations of the 5 biochars (n=5) | | | | | |
| --- | --- | --- | --- | --- | --- |
|  | FMB | CMB | PLB | PJB | CHB |
| P (g kg^-1^) | 42.7 | 25.4 | 23.7 | 4.59 | 0.62 |
| Al (g kg-1) | 17.6 | 12 | 5.78 | 1.28 | 0.59 |
| Fe(g kg-1) | 24.4 | 18.6 | 9.79 | 2.87 | 2.73 |
| Na (g kg^-1^) | 5.73 | 4.2 | 7.27 | 1.45 | 0.69 |
| Ca (g kg^-1^) | 32.8 | 21.3 | 34 | 18 | 3.75 |
| K (g kg^-1^) | 8.21 | 37.7 | 26.8 | 39.2 | 2.72 |
| Mg (g kg^-1^) | 28.9 | 16.7 | 10.9 | 3.67 | 0.94 |
| Zn (g kg^-1^) | 28.4 | 9.9 | 26.3 | 13.7 | 7.72 |
| Cd (mg kg^-1^) | 1.23 | 0.96 | 0.62 | <0.31 | <0.31 |
| Co (mg kg^-1^) | 16.4 | 11.2 | 6.25 | 1.41 | 2.05 |
| Cr (mg kg^-1^) | 39.5 | 26.5 | 13.5 | 3.68 | 2.16 |
| Cu (mg kg^-1^) | 214 | 182 | 101 | 32.3 | 40.1 |
| Ni (mg kg^-1^) | 84.4 | 30 | 26.3 | 8.35 | 7.7 |
| Pb (mg kg^-1^) | 502 | 295 | 528 | 214 | 271 |
|  |  |  |  |  |  |

| Table S3. Olsen-P, Exchangeable cations , CEC and NH_4_NO_3_ extractable (bioavailable) trace elements of the different biochars (n = 3) | | | | | |
| --- | --- | --- | --- | --- | --- |
|  | FMB | CMB | PLB | PJB | CHB |
| P (mg kg^-1^) | 1298 | 1437 | 607 | 383 | 28.1 |
| Ca (cmol_(+)_ kg^-1^) | 6.14 | 1.51 | 8.76 | 4.21 | 4.4 |
| K (cmol_(+)_ kg^-1^) | 1.61 | 43.9 | 31 | 59.6 | 1.6 |
| Na (cmol_(+)_ kg^-1^) | 5.62 | 1.05 | 2.83 | 0.23 | 0.25 |
| Mg (cmol_(+)_ kg^-1^) | 9.82 | 16.8 | 2.67 | 1.91 | 1.66 |
| CEC(cmol_(+)_ kg^-1^) | 23.2 | 63.3 | 45.3 | 65.9 | 7.91 |
| Zn (mg kg^-1^) | 12.4 | 165 | 172 | 1064 | 337 |
| Cd (mg kg^-1^) | 0.01 | 0.005 | 0.004 | 0.008 | 0.007 |
| Co (mg kg^-1^) | 0.022 | 0.017 | 0.02 | 0.012 | 0.011 |
| Cr (mg kg^-1^) | 0.01 | < 0.005 | < 0.005 | <0.002 | <0.002 |
| Cu (mg kg^-1^) | 0.067 | 0.204 | 0.04 | 0.007 | 0.006 |
| Mn (mg kg^-1^) | 5.62 | 3.25 | 4 | 1.74 | 10.4 |
| Ni (mg kg^-1^) | 0.032 | 0.027 | 0.017 | 0.06 | 0.021 |
| Pb (mg kg^-1^) | < 0.024 | < 0.025 | < 0.024 | 1.62 | <0.024 |

0

500

1000

1500

2000

2500

3000

3500

4000

4500

0.2

0.4

0.6

0.8

1.0

1.2

1.4

1.6

1433

1462

1038

1038

1270

1444

1596

1584

1700

1572

1467

3466

3442

3431

3419

3419

Absorbance

wave number (cm

-1

)

FM

CM

PL

CH

PJ

1441

Fig S1. FTIR spectra of FM, CM, PL, PJ, and CH biochars

**Selected images of the pot trials**


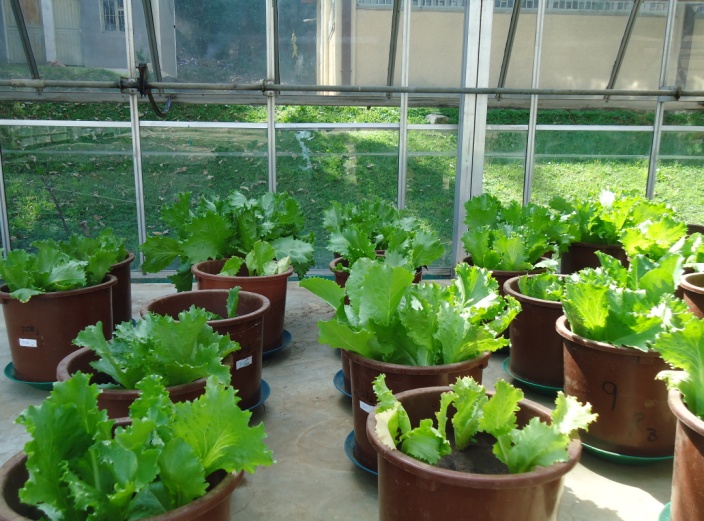

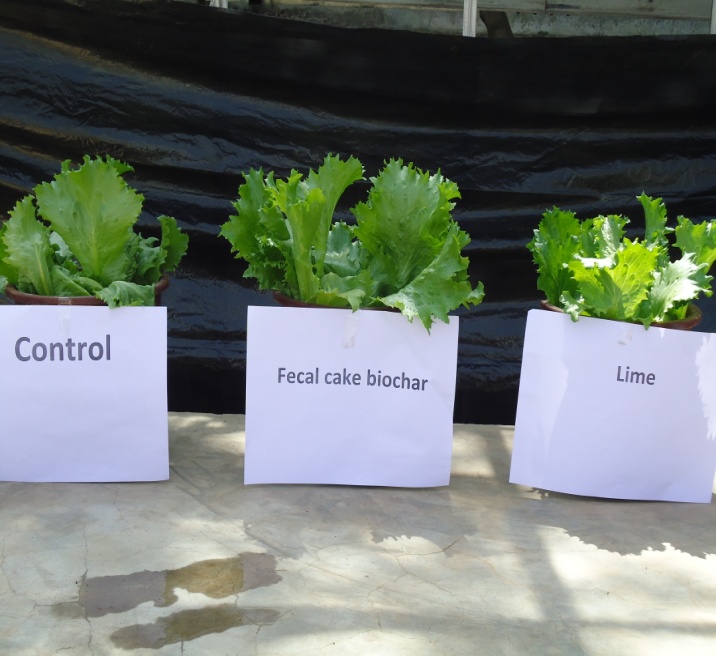


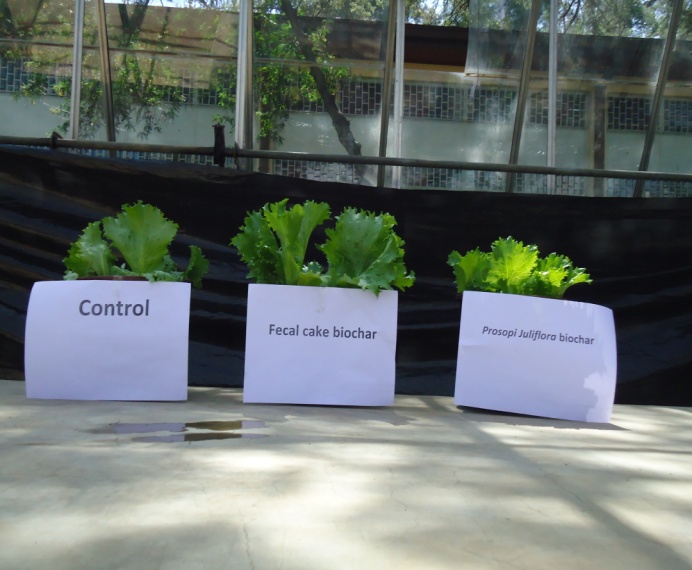

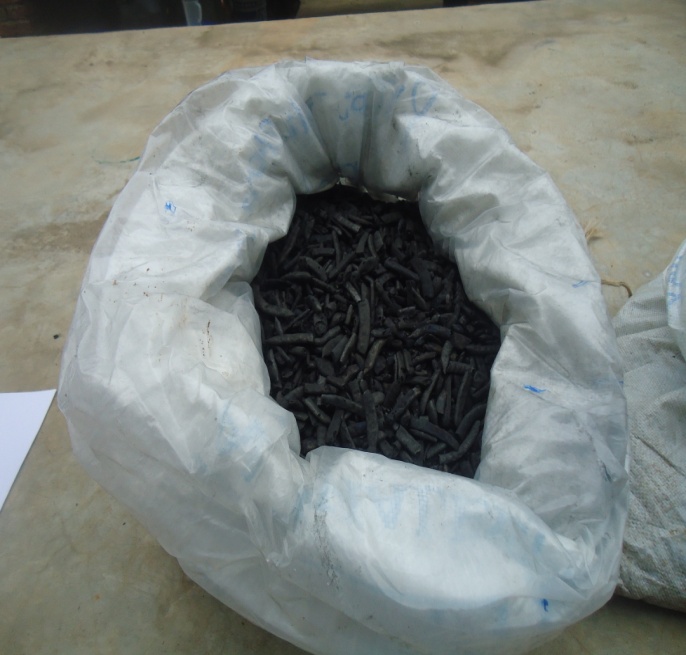


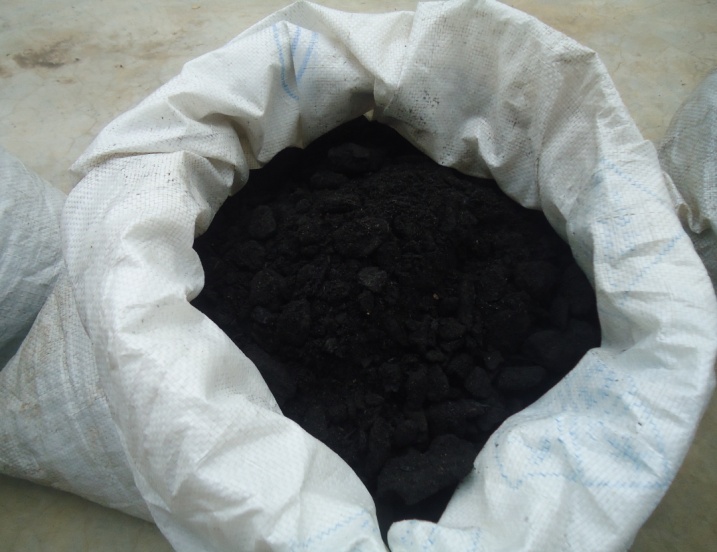

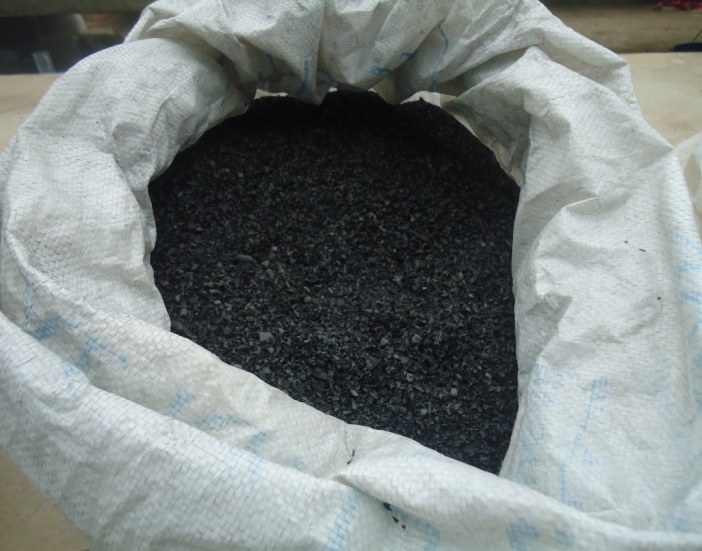

Supplement: Supplementary file 1 — 10.1186/s40064-016-2019-6 Chemical composition, surface and chemical properties of faecal matter, cow manure, poultry litter, prosopis juliflora pods, and coffee husk biochars. [file 40064_2016_2019_MOESM1_ESM.docx]
